# COVID-19 and risk of subsequent life-threatening secondary infections: a matched cohort study in UK Biobank

**DOI:** 10.1186/s12916-021-02177-0

**Published:** 2021-11-16

**Authors:** Can Hou, Yihan Hu, Huazhen Yang, Wenwen Chen, Yu Zeng, Zhiye Ying, Yao Hu, Yajing Sun, Yuanyuan Qu, Magnús Gottfreðsson, Unnur A. Valdimarsdóttir, Huan Song

**Affiliations:** 1 West China Biomedical Big Data Center, West China Hospital, Sichuan University, Guo Xue Lane 37#, Chengdu, 610041 China; 2 Med-X Center for Informatics, Sichuan University, Chengdu, China; 3 Division of Nephrology, Kidney Research Institute, State Key Laboratory of Biotherapy and Cancer Center, West China Hospital, Sichuan University, Chengdu, China; 4 Department of Internal Medicine, Faculty of Medicine, School of Health Sciences, University of Iceland, Reykjavík, Iceland; 5 Department of Infectious Diseases, Landspítali University Hospital, Reykjavik, Iceland; 6 Center of Public Health Sciences, Faculty of Medicine, University of Iceland, Reykjavík, Iceland; 7 Department of Medical Epidemiology and Biostatistics, Karolinska Institutet, Stockholm, Sweden; 8 Department of Epidemiology, Harvard T H Chan School of Public Health, Boston, MA USA

**Keywords:** COVID-19, Life-threatening infections, Severe secondary infections, Sepsis

## Abstract

**Background:**

With the increasing number of people infected with and recovered from coronavirus disease 2019 (COVID-19), the extent of major health consequences of COVID-19 is unclear, including risks of severe secondary infections.

**Methods:**

Based on 445,845 UK Biobank participants registered in England, we conducted a matched cohort study where 5151 individuals with a positive test result or hospitalized with a diagnosis of COVID-19 were included in the exposed group. We then randomly selected up to 10 matched individuals without COVID-19 diagnosis for each exposed individual (*n* = 51,402). The life-threatening secondary infections were defined as diagnoses of severe secondary infections with high mortality rates (i.e., sepsis, endocarditis, and central nervous system infections) from the UK Biobank inpatient hospital data, or deaths from these infections from mortality data. The follow-up period was limited to 3 months after the initial COVID-19 diagnosis. Using a similar study design, we additionally constructed a matched cohort where exposed individuals were diagnosed with seasonal influenza from either inpatient hospital or primary care data between 2010 and 2019 (6169 exposed and 61,555 unexposed individuals). After controlling for multiple confounders, Cox models were used to estimate hazard ratios (HRs) of life-threatening secondary infections after COVID-19 or seasonal influenza.

**Results:**

In the matched cohort for COVID-19, 50.22% of participants were male, and the median age at the index date was 66 years. During a median follow-up of 12.71 weeks, the incidence rate of life-threatening secondary infections was 2.23 (123/55.15) and 0.25 (151/600.55) per 1000 person-weeks for all patients with COVID-19 and their matched individuals, respectively, which corresponded to a fully adjusted HR of 8.19 (95% confidence interval [CI] 6.33–10.59). The corresponding HR of life-threatening secondary infections among all patients with seasonal influenza diagnosis was 4.50, 95% CI 3.34–6.08 (*p* for difference < 0.01). Also, elevated HRs were observed among hospitalized individuals for life-threatening secondary infections following hospital discharge, both in the COVID-19 (HR = 6.28 [95% CI 4.05–9.75]) and seasonal influenza (6.01 [95% CI 3.53–10.26], *p* for difference = 0.902) cohorts.

**Conclusion:**

COVID-19 patients have increased subsequent risks of life-threatening secondary infections, to an equal extent or beyond risk elevations observed for patients with seasonal influenza.

**Supplementary Information:**

The online version contains supplementary material available at 10.1186/s12916-021-02177-0.

## Background

As of August 2021, there are over 219 million cases of coronavirus disease 2019 (COVID-19) around the world, caused by severe acute respiratory syndrome coronavirus 2 (SARS-CoV-2) [1], with unprecedented impact on public health and the economy. Although severe COVID-19 cases were characterized by high case fatality, the majority (~ 81%) of COVID-19 patients only experienced flu-like symptoms and spontaneously recovered without specific medical interventions [2].

With more than 195 million people having recovered from COVID-19 [1], concerns regarding the short-term and long-term health consequences of COVID-19 are rising [3]. Previous studies have revealed that patients with viral respiratory infections may suffer an increased risk of secondary infections, including severe secondary infections with high mortality [4]. The possible underlying mechanisms for this phenomenon include direct damage of the upper airway and lungs due to viral-related inflammation, as well as virus-induced immunological impairment [5]. Therefore, it is plausible that COVID-19 can also alter people’s susceptibility to other severe secondary infections. This hypothesis is supported by a recent finding suggesting a profound impact of SARS-CoV-2 on immune systems [6], which seemed sustained even among individuals who have recovered from the primary infection [7, 8]. The hypothesis is also supported by epidemiology studies showing a high risk of severe secondary infections among patients hospitalized with COVID-19 [9–11]. Meanwhile, a recent longitudinal study further found that hospitalized COVID-19 patients had a higher risk of sepsis compared with hospitalized influenza patients [12]. Nevertheless, as previous studies were generally conducted in hospital-based settings, it remains unknown whether such elevated risk of severe secondary infections is present among mild COVID-19 cases and severe COVID-19 patients after hospital discharge. Leveraging data from UK Biobank which provides enriched data on sociodemographic, lifestyle, and medical factors, as well as timely updates of COVID-19-related outcomes, we conducted a matched cohort study to elucidate the association between COVID-19 and subsequent risk of life-threatening secondary infections. By additionally using seasonal influenza as a control of the “exposure” condition, we explored to what extent the observed associations were exclusive to COVID-19.

## Methods

### Study design

The UK Biobank is a longitudinal prospective cohort study that enrolled 502,507 participants aged between 40 and 69 years across the UK between 2006 and 2010 [13]. Detailed information on sociodemographic characteristics, lifestyle factors, and body measurements were collected at baseline. Through regular linkage to the Hospital Episode Statistics database, the Scottish Morbidity Record, and the Patient Episode Database for Wales, inpatient hospital data for UK Biobank participants registered in England, Scotland, and Wales were obtained, respectively. Mortality data were extracted from the National Health Service (NHS) Digital and NHS Central Register. Both inpatient hospital data and mortality data are deemed to reach a full coverage of UK Biobank participants from 1997 onwards [13]. Since the outbreak of COVID-19 in the UK, the linkage between UK Biobank and Public Health England’s (PHE) Second Generation Surveillance System has been established [14], for getting results of COVID-19 tests in English clinical diagnostics laboratories (based on RT-PCR of nose/throat swab samples, available since March 16, 2020). Meanwhile, UK Biobank also released updated primary care data obtained from two major general practice (GP) data system suppliers in England (EMIS and TPP), which covered approximately 409,000 (~ 92%) UK Biobank participants in England [15], for the purpose of facilitating COVID-19-related research. The diagnoses in both inpatient hospital data and primary care data in England have been widely validated in previous studies, showing consistently high quality and diagnostic accuracy [16, 17].

### Identification of COVID-19 and seasonal influenza

We identified COVID-19 cases by a positive result of COVID-19 test from the PHE (including tests for both hospitalized and non-hospitalized individuals) or hospital admission with a diagnosis of COVID-19 (according to the International Classification of Diseases 10th edition [ICD-10]: U07.1 or U07.2) based on UK Biobank inpatient hospital data (Fig. S1). To ensure the inclusion of both mild and severe cases, the ascertainment of seasonal influenza was based on both the UK Biobank inpatient hospital data according to ICD-10 codes (J09–J11) and updated primary care data using the corresponding primary care codes (listed in Additional File 1: Table S1-S2).

### Matched cohorts for COVID-19 and for seasonal influenza

**Fig. 1:**
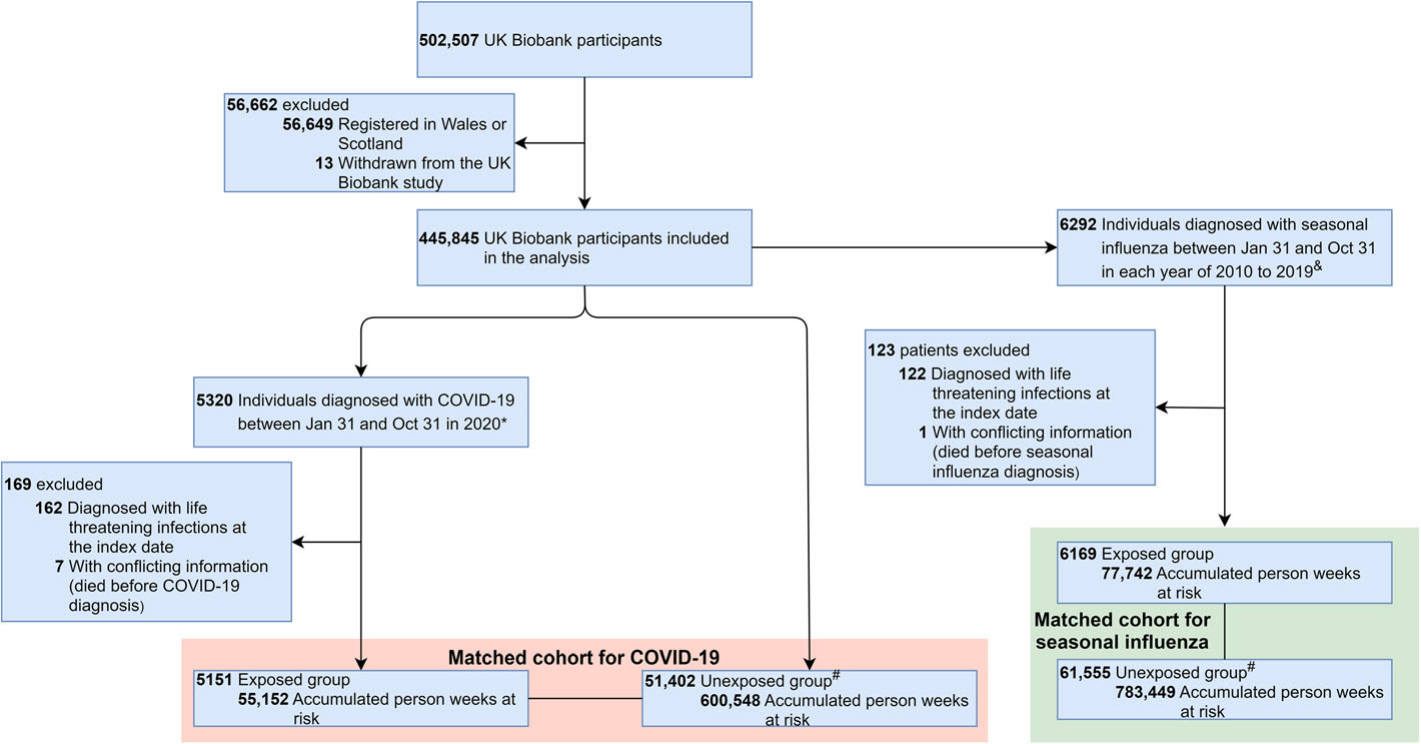
The flow chart of the matched cohorts for COVID-19 and for seasonal influenza. *January 31 is the date when the first UK COVID-19 case was confirmed; ^$^up to 10 individuals free of exposure disease at the index date were randomly selected and individually matched to each exposed individual by birth year, sex, decile of Townsend deprivation index, and Charlson comorbidity index (0, 1, 2, or 3+); ^&^individuals diagnosed with seasonal influenza between January 31 and October 31 from 2010 to 2019 based on primary care data and inpatient hospital data

In this matched cohort study, we constructed two matched cohorts. Specifically, after the exclusion of individuals who had withdrawn from the UK Biobank, the current study population was restricted to 445,845 UK Biobank participants registered in England (Fig. 1), as COVID-19 test results and follow-up data were unavailable for participants in Scotland and Wales. In the matched cohort for estimating the risk of life-threatening secondary infections after COVID-19 (i.e., the matched cohort for COVID-19), individuals diagnosed with COVID-19 between January 31 and October 31 in 2020 were included in the exposed group at the date of diagnosis. For comparison, we randomly selected up to 10 individuals (99.40% of exposed individuals had 10 matches, with a range of 1–10) without COVID-19 diagnosis at the diagnosis date of the index patient (i.e., index date) per COVID-19 patient, individually matched by birth year, sex, decile of Townsend deprivation index, and Charlson comorbidity index (CCI, 0, 1, 2, 3+).

Furthermore, to investigate whether the subsequent risk of life-threatening secondary infection is specific to COVID-19, we also constructed a matched cohort for seasonal influenza between 2010 and 2019. We included individuals diagnosed with seasonal influenza between January 31 and October 31 in each calendar year, as exposed individuals. For each seasonal influenza patient in each calendar year, we randomly selected up to 10 matched unexposed individuals (99.50% of exposed individuals had 10 matches, with a range of 1–10), using the same matching strategies as the matched cohort for COVID-19.

### Follow-up

Follow-up for all individuals started from the index date and ended on the date of death, the diagnosis date of life-threatening secondary infections, 3 months after the initial diagnosis, or the end of follow-up (i.e., December 31 of each calendar year), whichever occurred first. The follow-up of matched unexposed individuals was additionally censored if a corresponding exposure disease (e.g., COVID-19 for matched unexposed individuals in the matched cohort for COVID-19) was diagnosed during follow-up (about 1.0% of matched unexposed individuals were censored due to exposure diseases). The maximal surveillance period was set to 3 months because the majority of cases (3238/5151, 62.86%) in the matched cohort for COVID-19 were diagnosed in September or October 2020, with thereby limited follow-up time for studied outcomes, although biologically, the risk of life-threatening secondary infections may persist beyond this pre-defined follow-up period [18].

### Ascertainment of life-threatening secondary infections

We defined life-threatening secondary infections as severe secondary infections with high mortality rates (i.e., sepsis, endocarditis, and central nervous system infections) identified through a hospital admission with a diagnosis of these infections, or an underlying cause of death recorded as these infections (ICD-10 codes in the Additional File 1: Table S3), based on the UK Biobank inpatient hospital and mortality data. Particularly, we specifically excluded ICD-10 codes implying a viral infection for outcome identification (Additional File 1: Table S3).

### Covariates

Sociodemographic information (birth year, sex, and education level) and lifestyle factors (smoking status) were collected at recruitment using questionnaires. Townsend deprivation index, which is widely used as a measure of population-level deprivation [19], was assigned to each participant based on the postal codes provided at the baseline, with higher index scores denoting more deprivation. Body mass index (BMI) was assessed from height and weight measured at the baseline. Since somatic comorbidities may affect susceptibility to infectious disease, we calculated the CCI at the index date for each participant, as an index of baseline comorbidity level, according to diagnoses of the UK Biobank inpatient hospital data (ICD-10 codes listed in the Additional File 1: Table S4). In addition, to enable the consideration of baseline susceptibility to these studied life-threatening secondary infections, we extracted information about the history of life-threatening infections, defined as hospital admission with any diagnosis of these life-threatening infections in the UK Biobank inpatient hospital data prior to the index date.

### Statistical analysis

In both matched cohorts for COVID-19 and for seasonal influenza, hazard ratio (HR) with 95% confidence interval (CI), derived from the conditional Cox regression models, was used to estimate the association between the viral diseases (i.e., COVID-19 or seasonal influenza) and subsequent risk of life-threatening secondary infections. The Cox models were stratified by matching identifier (sex, birth year, CCI, and decile of Townsend deprivation index) [20] and adjusted for education level, Townsend deprivation index (as a continuous variable), CCI (as a continuous variable), BMI, smoking status, and history of life-threatening infections. We first explored the temporal change of the studied associations through the calculation of HRs for each week of follow-up (i.e., from the 1st to 12th weeks after the index date) and visualized the changing pattern by plotting the simulated trend of HRs using one-dimensional smoothing spline. Then, as more pronounced HRs were observed for the first few weeks, in addition to the overall follow-up period, we did separate the analyses for the follow-up time within and beyond 3 weeks, where the proportional hazards assumption was examined by Schoenfeld’s residuals, suggesting no indication of violation. Due to the limited number of incident cases of studied secondary infections, we always implemented penalized partial likelihood approach in the Cox models, in order to stabilize the coefficients [21].

To investigate the potential role of viral disease severity on the studied outcome, we did subgroup analysis by both the presence of COVID-19/seasonal influenza-related hospital admission and the application of operations/procedures during the hospitalization period. The subgroups were exposed individuals (1) with hospital admission and operations/procedures, (2) with hospital admission only, and (3) with neither hospital admission nor operations/procedures, along with their matched unexposed individuals. Also, to address the concern that the observed life-threatening secondary infections were mainly caused by aggregative medical interventions or a direct continuation of the preexisting primary infection, we repeated the main analysis for hospitalized patients using a more stringent definition for the life-threatening secondary infections, where the infections occurred during the hospital stay of the prior viral disease (i.e., COVID-19 or seasonal influenza) were excluded and we additionally required there was no diagnosis of COVID-19/seasonal influenza at the time of the life-threatening infections (i.e., life-threatening infections following hospital discharge). Namely, COVID-19 or influenza patients that have been discharged were followed for a re-admission to the hospital due to a life-threatening infection. In this analysis, the start of follow-up was reset to the discharge date, along with their matched unexposed individuals.

We compared the HRs from matched cohort for COVID-19 and these from matched cohort for seasonal influenza using the *z*-test. In addition, to test the robustness of our results to the choice of surveillance periods, we conducted sensitivity analyses where the maximum follow-up time was set as 1 month and no limit, respectively. As some diagnosed life-threatening infection cases had unclear pathogen (i.e., sepsis of unclear pathogen), we conducted an additional sensitivity analysis specifically on bacterial sepsis. All the analyses were conducted using SciPy (version 1.4.1), statsmodels (version 0.11.1), and lifelines (version 0.25.2) in Python 3.8, with two-sided *p*-value< 0.05 considered statistically significant.

## Results

### Characteristics and follow-up of the individuals

As shown in Fig. 1, the matched cohort for COVID-19 included 5151 COVID-19 patients and 51,402 matched individuals who had no diagnosis of COVID-19 at the index date. The median age of the COVID-19 exposed individuals at the index date was 66 years, with a roughly equal sex distribution (Table 1). Compared with matched unexposed individuals, individuals with COVID-19 were more likely to have a history of life-threatening infections (10.99% vs 7.33%, *p* < 0.001) and be overweight (31.84% vs 26.54% for BMI ≥ 29.9, *p* < 0.001). Among a total of 274 identified incident life-threatening secondary infections, the vast majority (> 95.0%) were sepsis (Table 1). However, 38.46% of diagnosed sepsis cases had unclear pathogen (unspecified sepsis), while 61.15% had bacterial sepsis, in both exposed and matched unexposed groups (Additional File 1: Table S5). More specifically, we found the most frequently identified pathogens of bacterial sepsis among COVID-19 patients to be *Escherichia coli* (25.00%) and other Gram-negative organisms (10.53%, Additional File 1: Table S5). The matched cohort for seasonal influenza contained 6169 exposed individuals with 61,555 matched unexposed individuals. Compared to individuals in the matched cohort for COVID-19, participants in the matched cohort for seasonal influenza were at a relatively younger age at the index date (62.00 vs 66.00 years) and had a longer time of follow-up (13.04 vs 12.71 weeks).

**Table 1:** Basic characteristics of the individuals in the matched cohort for COVID-19 and seasonal influenza

Characteristics	Matched cohort for COVID-19		Matched cohort for seasonal influenza	
	COVID-19 patients (*N* = 5151)	Matched individuals^&^ (*N* = 51,402)	Seasonal influenza patients (*N* = 6169)	Matched individuals^&^ (*N* = 61,555)
Age at index date, years	66.00 (58.00–74.00)	66.00 (58.00–74.00)	62.00 (55.00–69.00)	62.00 (55.00–69.00)
Townsend deprivation index^*^	− 1.46 (− 3.24–1.76)	− 1.45 (− 3.22–1.75)	− 1.49 (− 3.32–1.49)	− 1.48 (− 3.32–1.49)
Charlson comorbidity index^#^	0.00 (0.00–2.00)	0.00 (0.00–2.00)	0.00 (0.00–1.00)	0.00 (0.00–1.00)
Follow-up time, weeks	11.86 (9.71–13.04)	12.71 (10.29–13.04)	13.04 (13.04–13.04)	13.04 (13.04–13.04)
Sex				
*Female*	2564 (49.78%)	25,592 (49.79%)	3733 (60.51%)	37,289 (60.58%)
*Male*	2587 (50.22%)	25,810 (50.21%)	2436 (39.49%)	24,266 (39.42%)
Education level				
*College or university degree*	1203 (23.35%)	16,330 (31.77%)	1813 (29.39%)	19,521 (31.71%)
*A levels/AS levels or equivalent*	476 (9.24%)	5710 (11.11%)	680 (11.02%)	6810 (11.06%)
*O levels/GCSEs/CSEs or equivalent*	1503 (29.18%)	14,080 (27.39%)	1700 (27.56%)	16,622 (27.00%)
*Other qualifications*	1838 (35.68%)	14,127 (27.48%)	1800 (29.18%)	17,228 (27.99%)
*Unknown*	131 (2.54%)	1155 (2.25%)	176 (2.85%)	1374 (2.23%)
Body mass index				
*< 24.1*	964 (18.71%)	12,253 (23.84%)	1504 (24.38%)	15,608 (25.36%)
*24.1–29.9*	2503 (48.59%)	25,173 (48.97%)	3034 (49.18%)	29,810 (48.43%)
*≥ 29.9*	1640 (31.84%)	13,644 (26.54%)	1564 (25.35%)	15,742 (25.57%)
*Unknown*	44 (0.85%)	332 (0.65%)	67 (1.09%)	395 (0.64%)
Smoking				
*Yes*	2491 (48.36%)	23,707 (46.12%)	2827 (45.83%)	27,336 (44.41%)
*No*	2621 (50.88%)	27,357 (53.22%)	3291 (53.35%)	33,831 (54.96%)
*Unknown*	39 (0.76%)	338 (0.66%)	51 (0.83%)	388 (0.63%)
History of life-threatening infections^$^				
*Yes*	566 (10.99%)	3767 (7.33%)	366 (5.93%)	2589 (4.21%)
*No*	4585 (89.01%)	47,635 (92.67%)	5803 (94.07%)	58,966 (95.79%)
Life-threatening secondary infections diagnosis				
*Sepsis*	119 (96.75%)	141 (93.38%)	69 (97.18%)	133 (97.08%)
*Endocarditis*	3 (2.44%)	6 (3.97%)	0 (0.00%)	2 (1.46%)
*Central nervous system infections*	1 (0.81%)	4 (2.65%)	2 (2.82%)	2 (1.46%)

The values were reported as median (lower quantile-upper quantile) for continuous variables or number (%) for categorical variables; these variables were ascertained at the time of enrollment into the UK Biobank study: Townsend deprivation index, education level, BMI, and smoking

*Abbreviations*: *CSE*, Certificate of Secondary Education; *GCSE*, General Certificate of Secondary Education

*Townsend deprivation index was assigned to each individual based on their postcode location, and a greater index score implies a greater degree of deprivation

^#^Charlson comorbidity index at the index date, calculated based on inpatient hospital data (see Additional File 1: Table S4 for details)

^&^Up to 10 individuals free of exposure disease at the index date were randomly selected and individually matched to each exposed individual by birth year, sex, decile of Townsend deprivation index, and Charlson comorbidity index (0, 1, 2, or 3+)

^$^Defined as hospital admission with any diagnosis of life-threatening infections prior to the index date

### Risk of subsequent life-threatening secondary infections

**Fig. 2:**
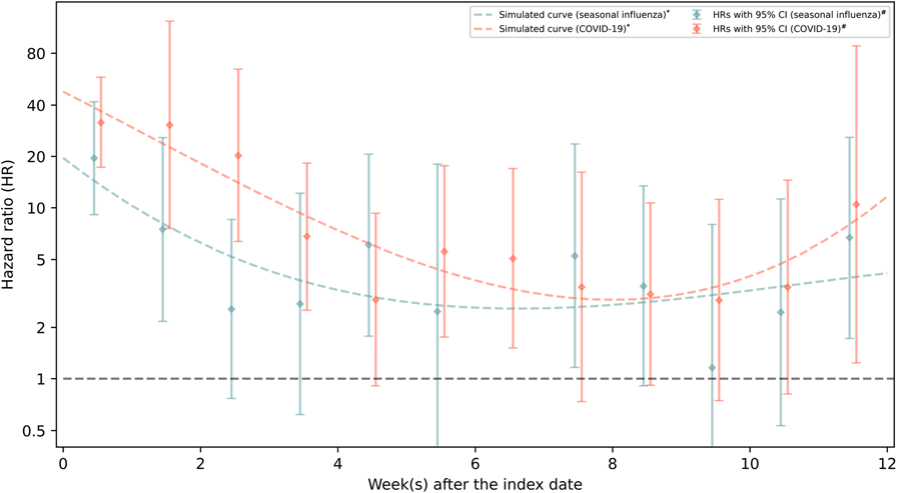
Temporal change of the associations between COVID-19/seasonal influenza and subsequent risk of life-threatening secondary infections. *The simulated curves were constructed using one-dimensional smoothing spline; ^#^Cox models were stratified by matching identifier (sex, birth year, Charlson comorbidity index, and decile of Townsend deprivation index) and adjusted for education level, Townsend deprivation index (as a continuous variable), Charlson comorbidity index (as a continuous variable), BMI, smoking status, and history of life-threatening infections

**Fig. 3:**
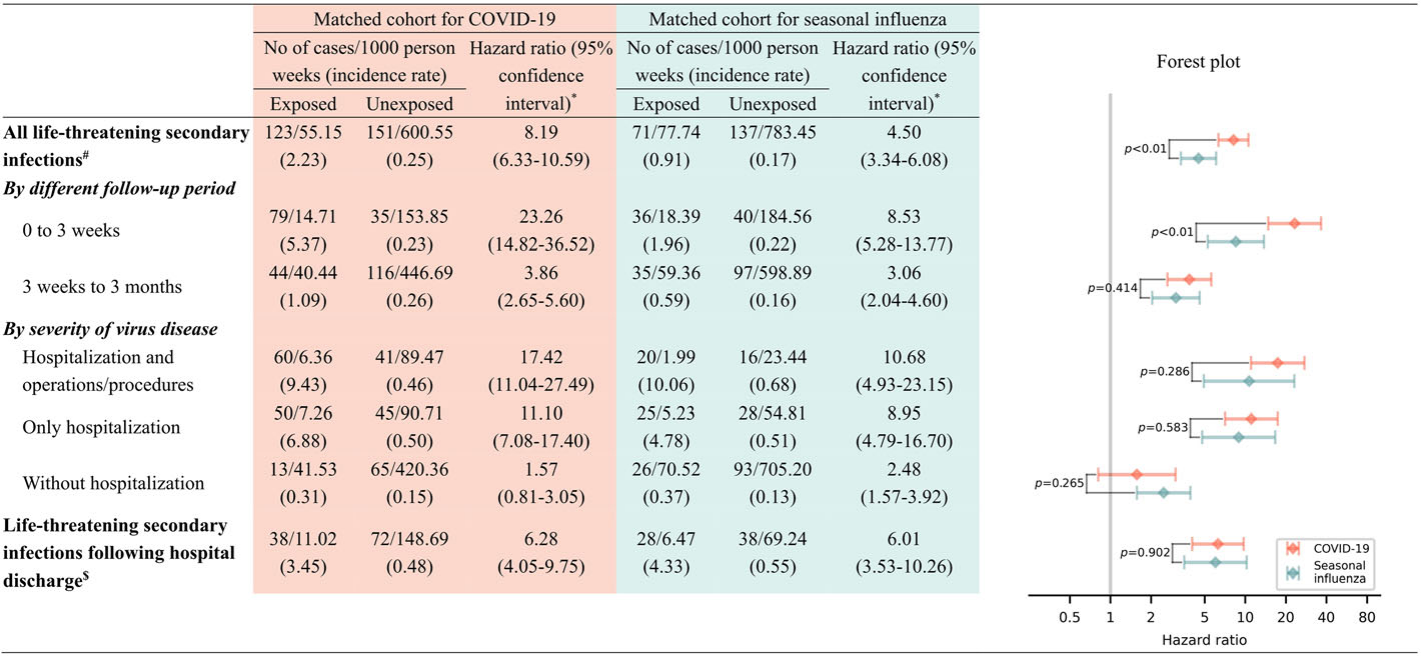
Comparison of the hazards of life-threatening secondary infections in COVID-19 and seasonal influenza patients. *Cox models were stratified by matching identifier (sex, birth year, Charlson comorbidity index, and decile of Townsend deprivation index) and adjusted for education level, Townsend deprivation index (as a continuous variable), Charlson comorbidity index (as a continuous variable), BMI, smoking status, and history of life-threatening infections; ^#^for all life-threatening secondary infections, the exposed individuals were stratified into three subgroups: with hospital admission and operations/procedures, with only hospital admission, and with neither hospital admission nor operations/procedures, along with their matched unexposed individuals; ^$^defined as having been discharged at the time of the life-threatening secondary infection diagnosis, and we also required that a diagnosis of prior virus infection was not present during the hospitalization of the subsequent severe secondary infections

In the matched cohort for COVID-19, we identified 123 incident life-threatening secondary infection cases in the COVID-19 exposed group and 151 in the matched unexposed group, during a median follow-up of 12.71 weeks. This corresponds to a crude incidence rate of 2.23 and 0.25 per 1000 person weeks in exposed and matched unexposed groups, respectively. Figure 2 shows the temporal patterns of the association between COVID-19 and life-threatening secondary infections, after adjusting for all important confounders. Briefly, the magnitude of the studied association was strongest in the first 3 weeks after the initial diagnosis of COVID-19, which then experienced an obvious decline but still remained significantly elevated until the 8th week after the viral infections. Correspondingly, with an average HR of 8.19 (95% CI 6.33–10.59) for the whole surveillance periods, the HR for < 3 weeks of follow-up and thereafter was 23.26 (95% CI 14.82–36.52) and 3.86 (95% CI 2.65–5.60, Fig. 3), respectively. Similar but less prominent HRs were observed based on the matched cohort for seasonal influenza, relative to these of the matched cohort for COVID-19 (Figs. 2 and 3).

By subtypes of COVID-19, the studied association was more pronounced for hospitalized COVID-19 patients, with the highest HR observed for those with operations/procedures experience (HR = 17.42, 95% CI 11.04–27.49). The HR was largely attenuated for COVID-19 cases without hospital admission (HR = 1.57, 95% CI 0.81–3.05). We further observed heightened HR for life-threatening secondary infections that is after hospital discharge (HR = 6.28 95% CI 4.05–9.75).

Results based on the matched cohort for seasonal influenza revealed a similar risk pattern with generally lower estimates, compared to those from matched cohort for COVID-19 (Fig. 3). The HR for all life-threatening secondary infections was 4.50 (95% CI 3.34–6.08), which was significantly lower than that after COVID-19 infection (*p* for difference < 0.01). Although it did not reach the level of significance, similar attenuation of HRs was observed for subgroups of hospitalized patients with seasonal influenza, with and without operations/procedures (*p* = 0.286 and 0.583, respectively), but not for seasonal influenza cases identified merely in primary care (*p* = 0.265).

In the sensitivity analyses, we observed largely comparable risk patterns after limiting the maximal follow-up period to 1 month or having no limit, both of which revealed significantly lower HRs of all life-threatening secondary infections after seasonal influenza than that after COVID-19 (Figs. S2 and S3, *p* for difference < 0.01 and *p* < 0.001, respectively), as the main analysis. Largely identical risk patterns were also observed in the sensitivity analysis on bacterial sepsis (Fig. S4).

## Discussion

In this matched cohort study based on the UK Biobank, we found that COVID-19 was associated with an overall increased risk of subsequent life-threatening secondary infections, after considering multiple important confounders such as socioeconomic status, comorbidities, and baseline susceptibility to infections. Although the excess risk was most pronounced among hospitalized COVID-19 patients who received invasive treatment during hospital care, patients who had been discharged (both due to COVID-19 and seasonal influenza) were also at an approximately 6-fold increased hazard of life-threatening secondary infections. Notably, although did not reach the level of significance, an approximately 1.5-fold increased hazard of severe secondary infections was also observed among mild (i.e., non-hospitalized) COVID-19 cases. Despite a general decrease with the time since the COVID-19 diagnosis, the association remained significant for at least 8 weeks after the initial diagnosis. Notably, we obtained similar or even higher HR of life-threatening secondary infections after COVID-19 compared to that after seasonal influenza. Apart from the varying disease severity, our results may suggest comparable or even more intense physiological alterations (e.g., immune dysregulation) as a result of COVID-19 compared to other severe viral respiratory infections, especially among hospitalized individuals. Given the high case fatality from the studied secondary infections and the huge and still increasing number of individuals affected by COVID-19, the disease burden caused by such problems could be substantial, highlighting the importance of continued follow-up of COVID-19 patients, even of patients who have been discharged from the hospital.

Our findings of an increased risk of developing life-threatening secondary infections among COVID-19 patients who have been discharged from hospital (i.e., recovering cases) and never required hospitalization (relatively mild cases) are novel, with no comparable data from other large-scale longitudinal investigations. Nevertheless, our results are corroborated by two recent prospective studies suggesting an association between COVID-19 and sepsis [11, 12], and cross-sectional studies suggesting a high incidence (> 50%) of sepsis among hospitalized COVID-19 patients [22]. Besides methodological shortcomings such as lack of comparable control group and no control for multiple important confounders, previous research has mainly focused on sepsis diagnosed during COVID-19 hospital treatment, with little effort to differentiate sepsis caused by COVID-19 from secondary sepsis induced by other pathogens. However, recent studies have revealed a general increase in the risk of secondary infections among COVID-19 patients. A recent meta-analysis summarized findings from retrospective case series studies, revealing a 14.3% prevalence of secondary bacterial infections among hospitalized COVID-19 patients [23]. This seemed comparable with a 12% reported prevalence of general secondary infections among affected patients during the 2009 H1N1 pandemic [24].

While the underlying mechanisms linking viral respiratory primary infection and subsequent secondary life-threatening infections remain unclear, it can be speculated that at least two biological pathways may potentially be involved in the process of predisposing COVID-19 patients to secondary infections. The first one highlights the damage of SARS-CoV-2 to epithelial cells in the respiratory tract [25], which may facilitate the binding of the endogenous and exogenous pathogens to cell surfaces, and consequently lead to the occurrence of secondary infections [26]. Furthermore, it is notable that COVID-19 can result in an immunocompromised state, induced, for example, by T cell activation/exhaustion [27]. This phenomenon is more frequently observed among COVID-19 patients with a more severe course [28], which may partially explain our results of further elevated risk of severe secondary infections among COVID-19 patients who experienced hospitalization or operations/procedures, as an indicator of the disease severity. Regarding the prolonged impact of viral infection on infection susceptibility, recent studies on the immune characteristics of COVID-19 patients suggested a continuation of immune dysregulation after the SARS-CoV-2 infection [7, 8], which is consistent with our findings that severe cases who had already been discharged from hospitals were still at approximately 6-fold increased hazard of getting life-threatening secondary infections, irrespective if they had been hospitalized for COVID-19 or seasonal influenza. Since the vast majority (~ 95%) of patients with COVID-19 are relatively mild cases [29], the potentially excess risk of life-threatening secondary infections within this population could be of great public health importance. Intriguingly, as similarly elevated risk was also observed for mild cases of seasonal influenza, such undifferentiated results may imply a common biological basis for the effect of these severe viral infections on the subsequent development of secondary severe infections. Furthermore, as we found significantly stronger associations between COVID-19 and life-threatening secondary infections, relative to seasonal influenza, mainly for the hospitalized individuals, our results imply that apart from a possibly higher degree of disease severity due to the general lack of pre-existing cross-reactive immunity to SARS-CoV-2 in the population, the physiological impact induced by severe SARS-CoV-2 infection may be more profound than that of seasonal influenza. However, this finding needs further investigations, ideally conducted among affected patients with COVID-19 and other respiratory viral diseases with comparable disease severity and optimally during the same study period, for better control of other relevant conditions to immune status (e.g., seasonal variation [30] and stress reaction to the pandemic [31]).

The strengths of the current study include the use of longitudinal data based on registers with full coverage of the UK Biobank participants in England, which minimized the selection bias and information bias. The timely updated data from hospital care and PHE enabled the analyses on the studied consequences of COVID-19 among affected patients with varied disease severity, including the ones with mild symptoms (i.e., non-hospitalized) or those having been discharged from hospital who have not been well investigated previously, with a relatively long follow-up period. In addition, by involving a matched cohort for seasonal influenza, our analysis enabled a comparison of the impact of two important viral infections on subsequent risk of severe secondary infections. Last, the availability of enriched data on sociodemographic information, lifestyle factors, and medical history also enabled considerations of several important confounders in the model.

One major concern regarding studies of this kind is that the identified life-threatening secondary infections can be directly induced by the studied viral infection (e.g., viral sepsis). This is particularly true for studies based on register data, since the detailed information for diagnoses and treatment are not available (i.e., about 38.0% of sepsis cases identified in our study were classified as “with unclear pathogens”). In the present study, to address this concern, we firstly excluded ICD-10 codes indicating viral infection for the outcome identification. Then, we performed a sub-analysis on life-threatening infections that did not occur during the COVID-19 hospitalization. Furthermore, we conducted a sensitivity analysis specifically focusing on bacterial sepsis. Although all these aforementioned analyses revealed a consistent and sustained increase in the risk of life-threatening secondary infections after COVID-19, this concern cannot be completely ruled out and should be taken into account when interpreting the results.

In addition, the comparability of disease severity between non-hospitalized COVID-19 and non-hospitalized seasonal influenza cases can be debated due to the different approaches for disease ascertainment. During the COVID-19 pandemic, a substantial proportion of individuals with asymptomatic COVID-19 got diagnosed [32], whereas seasonal influenza patients identified through primary care in the past years were mainly symptom-driven cases. Such difference may help explain the undifferentiated estimates for COVID-19 and seasonal influenza among exposed individuals without hospitalization. Furthermore, it is worth mentioning that the majority of identified seasonal influenza cases were diagnosed on clinical grounds, which can be nonspecific. Other notable limitations of our study include the small number of identified cases with the studied life-threatening secondary infections, leading to a limited statistical power to detect a differential impact of COVID-19 across the different severity levels of the primary infection. Also, with a relatively short surveillance period for most of the involved COVID-19 cases, studies investigating the risk of life-threatening secondary infections in a longer follow-up are warranted. Additionally, some important variables such as socioeconomic factors, BMI, and smoking status were only measured at recruitment of the UK Biobank and therefore may not reflect the individuals’ status at the time of diagnosis. Last, caution is needed when generalizing these results to a broader population due to some inherent limitations of the UK Biobank study, such as low response rate to study invitations (5.5%), and oversampling of the white population (i.e., 94.6% of individuals were white) [33].

## Conclusions

In this matched cohort study based on the UK Biobank, COVID-19 patients were at increased risk of subsequent life-threatening secondary infections, to an equal extent or even beyond risk elevations observed after seasonal influenza. Excess in such risks was also detected among severe patients after being discharged from the hospital. Thus, our findings highlight the importance of continued surveillance of patients with both current and prior severe COVID-19.

## Supplementary Information

**Additional file 1: Figures S1-S4 and Tables S1-S5. Figure S1.** Flow chart for the ascertainment of exposure and outcome diseases. **Figure S2.** Comparison of the hazards of life-threatening secondary infections in COVID-19 and seasonal influenza patients in the sensitivity analyses where the maximum follow-up time was set as 1 month. **Figure S3.** Comparison of the hazards of life-threatening secondary infections in COVID-19 and seasonal influenza patients in the sensitivity analyses where no limit was set to the maximum follow-up. **Figure S4.** Comparison of the hazards of bacteria sepsis in COVID-19 and seasonal influenza matched cohorts. **Table S1.** SNOMED CT codes used to ascertain seasonal influenza in primary care data (EMIS GP system) with mapping to 3-digits ICD-10 codes. **Table S2.** CTV3 codes used to ascertain seasonal influenza in primary care data (TPP GP system) with mapping to 3-digits ICD-10 codes. **Table S3.** ICD-10 codes used to ascertain major life-threatening infections. **Table S4.** ICD-10 coding algorithms for Charlson comorbidity index calculation. **Table S5.** Distribution of the underlying pathogens for sepsis cases among COVID-19/seasonal influenza patients and their matched individuals.

## Data Availability

Data from UK Biobank are available to all researchers upon making an application.

## Abbreviations

BMI: Body mass index
CCI: Charlson comorbidity index
CI: Confidence interval
COVID-19: Coronavirus disease 2019
GP: General practice
HR: Hazard ratio
ICD-10: International Classification of Diseases 10th edition
NHS: National Health Service
PHE: Public Health England
SARS-CoV-2: Severe acute respiratory syndrome coronavirus 2
