# Supplementary material for: COVID-19 and risk of subsequent life-threatening secondary infections: a matched cohort study in UK Biobank
Source: BMC Med. 2021 Nov 16;19:301. doi: 10.1186/s12916-021-02177-0 (PMC8592806; doi:10.1186/s12916-021-02177-0)
Supplement: Supplementary file 1 — Additional file 1: Figures S1-S4 and Tables S1-S5. Figure S1. Flow chart for the ascertainment of exposure and outcome diseases. Figure S2. Comparison of the hazards of life-threatening secondary infections in COVID-19 and seasonal influenza patients in the sensitivity analyses where the maximum follow-up time was set as 1 month. Figure S3. Comparison of the hazards of life-threatening secondary infections in COVID-19 and seasonal influenza patients in the sensitivity analyses where no limit was set to the maximum follow-up. Figure S4. Comparison of the hazards of bacteria sepsis in COVID-19 and seasonal influenza matched cohorts. Table S1. SNOMED CT codes used to ascertain seasonal influenza in primary care data (EMIS GP system) with mapping to 3-digits ICD-10 codes. Table S2. CTV3 codes used to ascertain seasonal influenza in primary care data (TPP GP system) with mapping to 3-digits ICD-10 codes. Table S3. ICD-10 codes used to ascertain major life-threatening infections. Table S4. ICD-10 coding algorithms for Charlson comorbidity index calculation. Table S5. Distribution of the underlying pathogens for sepsis cases among COVID-19/seasonal influenza patients and their matched individuals. [file 12916_2021_2177_MOESM1_ESM.docx]

**COVID-19 and risk of subsequent life-threatening secondary infections: a matched cohort study in UK Biobank**

**Additional File 1**

Can Hou; Yihan Hu; Huazhen Yang; Wenwen Chen; Yu Zeng; Zhiye Ying; Yao Hu; Yajing Sun; Yuanyuan Qu; Magnús Gottfreðsson; Unnur A Valdimarsdóttir; Huan Song

**Content**

[**Figure S1** Flow chart for the ascertainment of exposure and outcome diseases 2](#_Toc80194434)

[**Figure S2** Comparison of the hazards of life-threatening secondary infections in COVID-19 and seasonal influenza patients in the sensitivity analyses where the maximum follow-up time was set as 1 month 3](#_Toc80194435)

[**Figure S3** Comparison of the hazards of life-threatening secondary infections in COVID-19 and seasonal influenza patients in the sensitivity analyses where no limit was set to the maximum follow-up 5](#_Toc80194436)

[**Figure S4** Comparison of the hazards of bacteria sepsis in COVID-19 and seasonal influenza matched cohorts 7](#_Toc80194437)

[**Table S1** SNOMED CT codes used to ascertain seasonal influenza in primary care data (EMIS GP system) with mapping to 3-digits ICD-10 codes 9](#_Toc80103914)

[**Table S2** CTV3 codes used to ascertain seasonal influenza in primary care data (TPP GP system) with mapping to 3-digits ICD-10 codes 13](#_Toc80103915)

[**Table S3** ICD-10 codes used to ascertain major life-threatening infections 15](#_Toc80103916)

[**Table S4** ICD-10 coding algorithms for Charlson comorbidity index calculation 16](#_Toc80103917)

[**Table S5** Distribution of the underlying pathogens for sepsis cases among COVID-19/seasonal influenza patients and their matched individuals 17](#_Toc80103918)

**
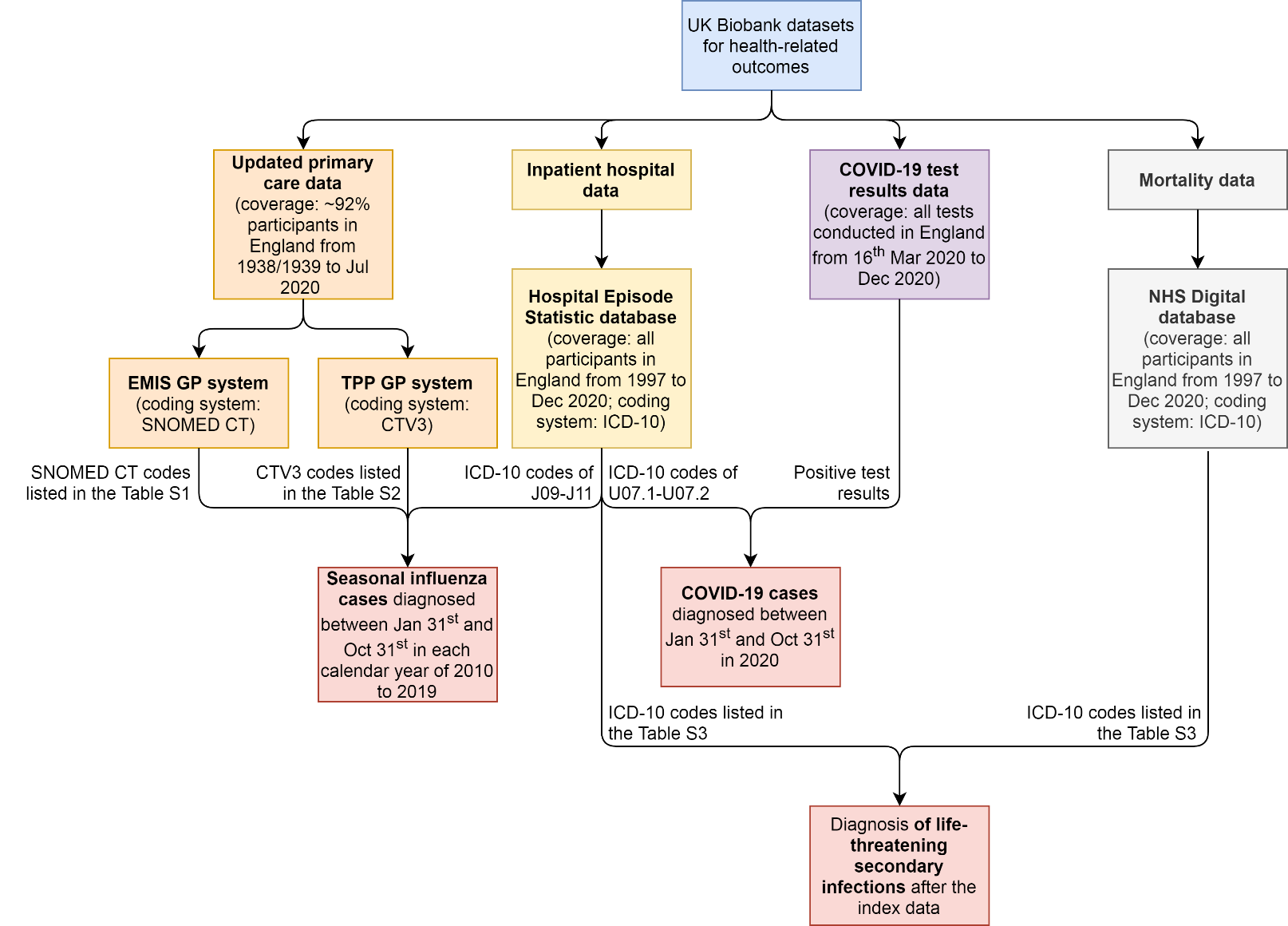
**

**Figure S1** Flow chart for the ascertainment of exposure and outcome diseases

|  | Matched cohort for COVID-19 | | | Matched cohort for seasonal influenza | | | Forest plot |
| --- | --- | --- | --- | --- | --- | --- | --- |
|  | No of cases/1,000 person weeks (incidence rate) | | Hazard ratio (95% confidence interval)^*^ | No of cases/1,000 person weeks (incidence rate) | | Hazard ratio (95% confidence interval)^*^ |  |
|  | Exposed | Unexposed |  | Exposed | Unexposed |  |  |
| **All life-threatening secondary infections^#^** | 88/21.06 (4.18) | 52/222.78 (0.23) | 16.05  (11.09-23.23) | 41/26.61 (1.54) | 55/267.35 (0.21) | 6.77  (4.38-10.45) | 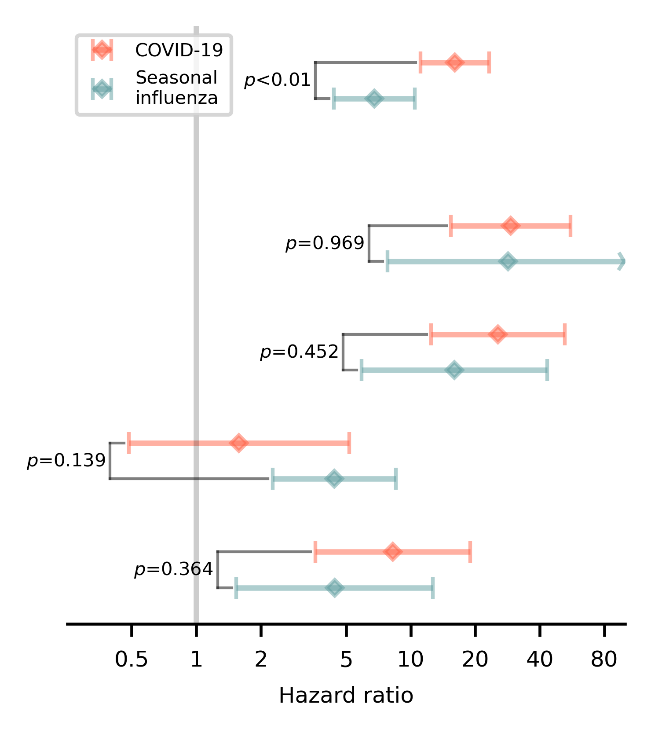 |
| ***By severity of virus disease*** |  |  |  |  |  |  |  |
| Hospitalization and  operations/procedures | 47/2.50 (18.81) | 16/31.00 (0.52) | 29.26  (15.41-55.56) | 12/0.73 (16.54) | 9/7.91  (1.14) | 28.44  (7.80-103.73) |  |
| Only hospitalization | 37/2.73 (13.56) | 13/32.12 (0.40) | 25.58  (12.48-52.47) | 14/1.79 (7.83) | 12/18.42 (0.65) | 15.97  (5.90-43.24) |  |
| Without hospitalization | 4/15.84 (0.25) | 23/159.67 (0.14) | 1.58  (0.48-5.16) | 15/24.10 (0.62) | 34/241.02 (0.14) | 4.40  (2.27-8.52) |  |
| **Life-threatening secondary infections following hospital discharge^$^** | 13/2.87 (4.53) | 18/37.65 (0.48) | 8.24  (3.59-18.92) | 7/1.89 (3.71) | 15/19.59 (0.77) | 4.42  (1.54-12.71) |  |
|  | | | | | | |  |

**Figure S2** Comparison of the hazards of life-threatening secondary infections in COVID-19 and seasonal influenza patients in the sensitivity analyses where the maximum follow-up time was set as 1 month

^*^Cox models were stratified by matching identifier (sex, birth year, Charlson comorbidity index and decile of Townsend deprivation index) and adjusted for education level, Townsend deprivation index (as continuous variable), Charlson comorbidity index (as continuous variable), BMI, smoking status and history of life-threatening infections;

^#^for all life-threatening secondary infections, patients and their matched individuals were stratified into three subgroups: patients with hospital admissions and operations/procedures during the follow-up period; patients with only hospital admissions; and patients with no hospital admissions;

^$^defined as have been discharged at the time of the life-threatening secondary infections diagnosis. We also required that a diagnosis of prior virus infection was not present during the hospitalization of the subsequent severe secondary infections.

|  | Matched cohort for COVID-19 | | | Matched cohort for seasonal influenza | | | Forest plot |
| --- | --- | --- | --- | --- | --- | --- | --- |
|  | No of cases/1,000 person weeks (incidence rate) | | Hazard ratio (95% confidence interval)^*^ | No of cases/1,000 person weeks (incidence rate) | | Hazard ratio (95% confidence interval)^*^ |  |
|  | Exposed | Unexposed |  | Exposed | Unexposed |  |  |
| **All life-threatening secondary infections^#^** | 157/108.12 (1.45) | 358/1,244.35 (0.29) | 4.92  (4.00-6.04) | 114/208.40 (0.55) | 335/2,113.31 (0.16) | 2.93  (2.34-3.67) | 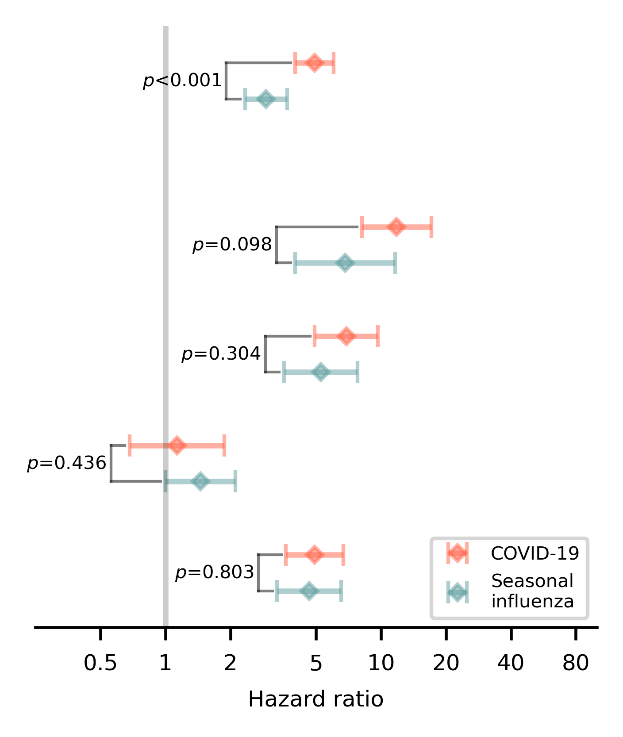 |
| ***By severity of virus disease*** |  |  |  |  |  |  |  |
| Hospitalization and  operations/procedures | 71/15.29 (4.64) | 89/238.33 (0.37) | 11.81  (8.15-17.12) | 32/5.59 (5.73) | 37/72.53 (0.51) | 6.83  (4.00-11.64) |  |
| Only hospitalization | 67/18.09 (3.70) | 113/238.67 (0.47) | 6.90  (4.93-9.65) | 46/15.80 (2.91) | 83/172.85 (0.48) | 5.26  (3.55-7.79) |  |
| Without hospitalization | 19/74.73 (0.25) | 156/767.35 (0.20) | 1.13  (0.68-1.88) | 36/187.02 (0.19) | 215/1,867.94 (0.12) | 1.45  (1.00-2.11) |  |
| **Life-threatening secondary infections following hospital discharge^$^** | 70/31.01 (2.26) | 189/442.94 (0.43) | 4.92  (3.62-6.69) | 62/20.66 (3.00) | 112/233.36 (0.48) | 4.64  (3.29-6.54) |  |
|  | | | | | | |  |

**Figure S3** Comparison of the hazards of life-threatening secondary infections in COVID-19 and seasonal influenza patients in the sensitivity analyses where no limit was set to the maximum follow-up

^*^Cox models were stratified by matching identifier (sex, birth year, Charlson comorbidity index and decile of Townsend deprivation index) and adjusted for education level, Townsend deprivation index (as continuous variable), Charlson comorbidity index (as continuous variable), BMI, smoking status and history of life-threatening infections;

^#^for all life-threatening secondary infections, patients and their matched individuals were stratified into three subgroups: patients with hospital admissions and operations/procedures during the follow-up period; patients with only hospital admissions; and patients with no hospital admissions;

^$^defined as have been discharged at the time of the life-threatening secondary infections diagnosis. We also required that a diagnosis of prior virus infection was not present during the hospitalization of the subsequent severe secondary infections.

|  | Matched cohort for COVID-19 | | | Matched cohort for seasonal influenza | | | Forest plot |
| --- | --- | --- | --- | --- | --- | --- | --- |
|  | No of cases/1,000 person weeks (incidence rate) | | Hazard ratio (95% confidence interval)^*^ | No of cases/1,000 person weeks (incidence rate) | | Hazard ratio (95% confidence interval)^*^ |  |
|  | Exposed | Unexposed |  | Exposed | Unexposed |  |  |
| **All Bacterial sepsis^#^** | 76/55.16 (1.38) | 83/600.56 (0.14) | 9.60  (6.80-13.56) | 44/77.76 (0.57) | 80/783.46 (0.10) | 4.91  (3.31-7.28) | 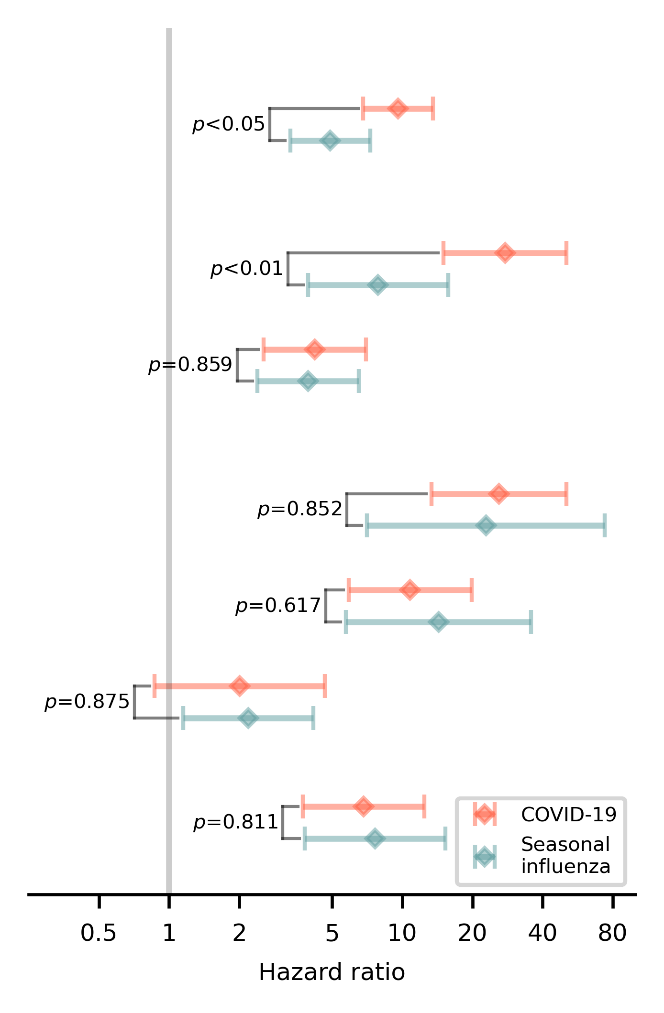 |
| ***By different follow-up period*** |  |  |  |  |  |  |  |
| 0 to 3 weeks | 50/14.76 (3.39) | 22/153.87 (0.14) | 27.60  (15.03-50.68) | 18/18.42 (0.98) | 24/184.58 (0.13) | 7.88  (3.95-15.72) |  |
| 3 weeks to 3 months | 26/40.66 (0.64) | 61/446.95 (0.14) | 4.21  (2.54-6.99) | 26/59.55 (0.44) | 56/599.22 (0.09) | 3.95  (2.40-6.51) |  |
| ***By severity of virus disease*** |  |  |  |  |  |  |  |
| Hospitalization and  operations/procedures | 39/6.36 (6.13) | 21/89.48 (0.23) | 25.97  (13.32-50.64) | 15/1.99 (7.55) | 10/23.44 (0.43) | 22.85  (7.06-73.99) |  |
| Only hospitalization | 28/7.27 (3.85) | 27/90.71 (0.30) | 10.81  (5.89-19.84) | 16/5.23 (3.06) | 14/54.82 (0.26) | 14.31  (5.73-35.72) |  |
| Without hospitalization | 9/41.53 (0.22) | 35/420.37 (0.08) | 2.01  (0.87-4.65) | 13/70.54 (0.18) | 56/705.20 (0.08) | 2.19  (1.15-4.16) |  |
| **Bacterial sepsis following hospital discharge^$^** | 22/10.95 (2.01) | 37/148.70 (0.25) | 6.83  (3.75-12.42) | 19/6.48 (2.93) | 20/69.44 (0.29) | 7.64  (3.81-15.31) |  |
|  | | | | | | |  |

**Figure S4** Comparison of the hazards of bacteria sepsis in COVID-19 and seasonal influenza matched cohorts

^*^Cox models were stratified by matching identifier (sex, birth year, Charlson comorbidity index and decile of Townsend deprivation index) and adjusted for education level, Townsend deprivation index (as continuous variable), Charlson comorbidity index (as continuous variable), BMI, smoking status and history of life-threatening infections;

^#^patients and their matched individuals were stratified into three subgroups: patients with hospital admissions and operations/procedures during the follow-up period; patients with only hospital admissions; and patients with no hospital admissions;

^$^defined as have been discharged at the time of the bacterial sepsis diagnosis. We also required that a diagnosis of prior virus infection was not present during the hospitalization of the subsequent bacterial sepsis.

**Table S1** SNOMED CT codes used to ascertain seasonal influenza in primary care data (EMIS GP system) with mapping to 3-digits ICD-10 codes

| **ICD-10** | | **SNOMED CT** | |
| --- | --- | --- | --- |
| **Code** | **Description** | **Code^*^** | **Description** |
| J09 | Influenza due to identified zoonotic or pandemic influenza virus | 46171006 | Influenza due to Influenza virus, type A, porcine |
|  |  | 55604004 | Avian influenza |
|  |  | 195920000 | Influenza with pneumonia, influenza virus identified |
|  |  | 195922008 | Influenza with other respiratory manifestation |
|  |  | 196200002 | [X]Influenza with other respiratory manifestations, influenza virus identified |
|  |  | 427873006 | Influenza due to influenza virus type A, avian, H5N1 strain (disorder) |
|  |  | 450715004 | Influenza due to Influenza A virus subtype H7 (disorder) |
|  |  | 450716003 | Influenza due to Influenza A virus subtype H9 |
|  |  | 707448003 | Influenza due to Influenza A virus subtype H7N9 (disorder) |
|  |  | 711128004 | Influenza due to influenza virus type A, avian, H3N2 strain |
|  |  | 719865001 | Influenza caused by pandemic influenza virus (disorder) |
|  |  | 772828001 | Influenza caused by Influenza A virus subtype H5N1 (disorder) |
|  |  | 142921000119103 | Upper respiratory symptoms due to avian influenza |
|  |  | 143111000119103 | Pneumonia due to avian influenza (disorder) |
|  |  | 616171000000100 | Influenza with other respiratory manifestation (disorder) |
|  |  | 1033051000000101 | Influenza due to zoonotic influenza virus (disorder) |
|  |  | 1033071000000105 | Influenza due to pandemic influenza virus |
|  |  | 10629191000119100 | Bronchiolitis caused by influenza virus |
|  |  | 10685111000119102 | Upper respiratory tract infection due to Influenza (disorder) |
| J10 | Influenza due to identified seasonal influenza virus | 6142004 | Influenza |
|  |  | 24662006 | Influenza due to Influenza virus, type B |
|  |  | 42964004 | Influenza with pneumonia |
|  |  | 43692000 | Influenzal acute upper respiratory infection |
|  |  | 63039003 | Influenza with respiratory manifestation other than pneumonia |
|  |  | 78431007 | Influenza due to Influenza virus, type A, human |
|  |  | 81524006 | Influenza due to Influenza virus, type C |
|  |  | 442438000 | Influenza due to Influenza A virus (disorder) |
|  |  | 442696006 | Influenza A (H1N1) |
|  |  | 713083002 | Influenza caused by Influenza A virus subtype H5 (disorder) |
|  |  | 719590007 | Influenza caused by seasonal influenza virus (disorder) |
|  |  | 772810003 | Influenza caused by Influenza A virus subtype H3N2 (disorder) |
|  |  | 142931000119100 | Pneumonia due to H1N1 influenza |
|  |  | 142941000119109 | Upper respiratory tract infection due to H1N1 influenza (disorder) |
|  |  | 328531000119104 | Upper respiratory tract infection due to Influenza A (disorder) |
|  |  | 418181000000104 | [X]Influenza with other respiratory manifestations, influenza virus identified (disorder) |
|  |  | 1033091000000109 | Influenza due to seasonal influenza virus (disorder) |
|  |  | 1033111000000104 | Influenza with pneumonia due to seasonal influenza virus (disorder) |
| J11 | Influenza, virus not identified | 41269000 | Influenzal bronchopneumonia |
|  |  | 95891005 | Influenza-like illness |
|  |  | 195921001 | Influenza with pneumonia NOS |
|  |  | 195925005 | Influenza with respiratory manifestations NOS |
|  |  | 195931008 | (Influenza like illness) or (influenza NOS) |
|  |  | 196202005 | [X]Influenza with other respiratory manifestations, virus not identified |
|  |  | 266353003 | Influenza NOS |
|  |  | 772839003 | Pneumonia caused by Influenza A virus (disorder) |
|  |  | 16311000119108 | Pneumonia due to influenza |
|  |  | 430891000000103 | [X]Influenza with other respiratory manifestations, virus not identified (disorder) |
|  |  | 616161000000107 | Influenza with pneumonia NOS |
|  |  | 616181000000103 | Influenza with respiratory manifestations NOS (disorder) |
|  |  | 670551000000108 | Influenza NOS |
| Descriptions of SNOMED CT codes and their mapping to ICD-10 codes is based on UK SNOMED CT Clinical Edition (release SNOMEDCT2_31.0.0_20201028000001);  *For SNOMED CT codes that can be mapped to multiple ICD-10 codes within J09-J11, only the mapping with the highest priority is shown in this table;  Abbreviation: SNOMED CT: Systemized Nomenclature of Medicine Clinical Terms, GP: general practitioner, ICD-10: The International Statistical Classification of Diseases and Related Health Problems 10th Revision. | | | |

**Table S2** CTV3 codes used to ascertain seasonal influenza in primary care data (TPP GP system) with mapping to 3-digits ICD-10 codes

| **ICD-10** | | **CTV3** | |
| --- | --- | --- | --- |
| **Code** | **Description** | **Code** | **Description** |
| J09 | Influenza due to identified zoonotic or pandemic influenza virus | H27.. | Influenza |
|  |  | H2701 | Influenza with pneumonia, influenza virus identified |
|  |  | H271. | Influenza with other respiratory manifestation |
|  |  | Hyu04 | [X]Influenza with other respiratory manifestations, influenza virus identified |
|  |  | XaeVK | Influenza due to zoonotic influenza virus |
|  |  | XaeVL | Influenza due to pandemic influenza virus |
|  |  | XaMih | Avian influenza |
| J10 | Influenza due to identified seasonal influenza virus | H27.. | Influenza |
|  |  | H270. | Influenzal pneumonia |
|  |  | H2701 | Influenza with pneumonia, influenza virus identified |
|  |  | H271. | Influenza with other respiratory manifestation |
|  |  | Hyu04 | [X]Influenza with other respiratory manifestations, influenza virus identified |
|  |  | XaQQp | Influenza due to Influenza A virus subtype H1N1 |
|  |  | XaeVM | Influenza due to seasonal influenza virus |
|  |  | XaeVN | Influenza with pneumonia due to seasonal influenza virus |
| J11 | Influenza, virus not identified | H27.. | Influenza |
|  |  | H270. | Influenzal pneumonia |
|  |  | H2700 | Influenza with bronchopneumonia |
|  |  | H270z | Influenza with pneumonia NOS |
|  |  | H271. | Influenza with other respiratory manifestation |
|  |  | H271z | Influenza with respiratory manifestations NOS |
|  |  | H27z. | (Influenza like illness) or (influenza NOS) |
|  |  | Hyu06 | [X]Influenza with other respiratory manifestations, virus not identified |
|  |  | XE0YK | Influenza NOS |
|  |  | XM0rz | Influenza-like illness |
| Descriptions of CTV3 codes and their mapping to ICD-10 codes is based on the resources provided by The NHS Business Services Authority (http://biobank.ndph.ox.ac.uk/showcase/refer.cgi?id=592);  Abbreviation: CTV3: Clinical Terms Version 3, GP: general practitioner, ICD-10: The International Statistical Classification of Diseases and Related Health Problems 10th Revision. | | | |

**Table S3** ICD-10 codes used to ascertain major life-threatening infections

| **Diseases** | | **ICD-10 codes** |
| --- | --- | --- |
| Sepsis | Bacterial sepsis | A02.1, A04.0–A04.3, A39 (excl. A39.0, A39.1, A39.8, A39.9), A40–A41 (excl. A41.8, A41.9), A42.7, A48, A90, A91, B95–B99 (excl. B97) |
|  | Fungal sepsis | B37.7, B38.7, B39.3, B40.7, B41.7, B42.7, B44.7, B45.7, B46.4 |
|  | Sepsis of unclear pathogen | A41.8, A41.9 |
| Endocarditis | | I33, I38, I39 |
| Central nervous system infections | | A06.6, A17, A39.0, A39.8, A39.9, B37.5, B38.4, B43.1, B50.0, B58.2, B60.2, G00–G08 (excl. G02.0, G04.1, G05.1) |
| Reference: Song, Huan, et al. "Stress related disorders and subsequent risk of life threatening infections: population based sibling controlled cohort study." bmj 367 (2019): l5784. | | |

ICD-10 codes included in the original list but imply virus infection were excluded: A80–A89, A92-A99, B00.3, B00.4, B01.0, B01.1,B02.0, B02.1, B05.0, B05.1, B06.0, B22.0, B26.1, B26.2, B97, G02.0, G04.1 and G05.1.

**Table S4** ICD-10 coding algorithms for Charlson comorbidity index calculation

| **Score** | **Comorbidity** | **ICD-10 codes** |
| --- | --- | --- |
| 1 | Myocardial infarction | I21, I22, I25.2 |
| 1 | Congestive heart failure | I11.0, I13.0, I13.2, I50 |
| 1 | Peripheral vascular disease | I70, I71, I73.1, I73.8, I73.9, I77.1, I79.0, I79.2, K55.1, K55.8, K55.9, R02, Z95.8, Z95.9 |
| 1 | Cerebrovascular disease | G45, G46, I60, I61, I62, I63, I64, I65, I66, I67, I68, I69 |
| 1 | Dementia | F00, F01, F02, F03, F051, G30, G31.1 |
| 1 | Chronic pulmonary disease | J60, J61, J62, J63, J64, J65, J66, J67, J68.4, J70, J84.1, J92.0, J96.1, J98.2, J40, J41, J42, J43, J44, J45, J46, J47 |
| 1 | Connective tissue disease | M05, M06, M30, M31.5, M32, M33, M34, M35.1, M35.3, M36.0 |
| 1 | Ulcer disease | K25, K26, K27, K28 |
| 1 | Mild liver disease | B18, K70.0, K70.1, K70.2, K70.3, K70.9, K71.3, K71.4, K71.5, K71.7, K73, K74, K76.0, K76.2, K76.3, K76.4, K76.8, K76.9 |
| 1 | Diabetes mellitus | E10.0, E10.1, E10.6, E10.8, E10.9, E11.0, E11.1, E11.6, E11.8, E11.9, E12.0, E12.1, E12.6, E12.8, E12.9, E13.0, E13.1, E13.6, E13.8, E13.9, E14.0, E14.1, E14.6, E14.8, E14.9 |
| 2 | Hemiplegia | G04.1, G11.4, G80.1, G80.2, G81, G82, G83.9, G83.0, G83.1, G83.2, G83.3, G83.4 |
| 2 | Moderate/severe renal disease | I12.0, I13.1, N03.2, N03.3, N03.4, N03.5, N03.6, N03.7, N05.2, N05.3, N05.4, N05.5, N05.6, N05.7, N18, N19, N25.0, Z94.0, Z99.2 |
| 2 | Diabetes mellitus with chronic complications | E10.2, E10.3, E10.4, E10.5, E10.7, E11.7, E12.7, E13.7, E14.7, E11.2, E11.3, E11.4, E11.5, E12.2, E12.3, E12.4, E12.5, E13.2, E13.3, E13.4, E13.5, E14.2, E14.3, E14.4, E14.5 |
| 2 | Any tumor | C97, C43, C00, C01, C02, C03, C04, C05, C06, C07, C08, C09, C10, C11, C12, C13, C14, C15, C16, C17, C18, C19, C20, C21, C22, C23, C24, C25, C26, C30, C31, C32, C33, C34, C37, C38, C39, C40, C41, C45, C46, C47, C48, C49, C50, C51, C52, C53, C54, C55, C56, C57, C58, C60, C61, C62, C63, C64, C65, C66, C67, C68, C69, C70, C71, C72, C73, C74, C75, C76 |
| 2 | Leukemia | C91, C92, C93, C94, C95 |
| 2 | Lymphoma | C81, C82, C83, C84, C85, C88, C90, C96 |
| 3 | Moderate/severe liver disease | I85, K70.4, K72, K76.6 |
| 6 | Metastatic solid tumor | C77, C78, C79, C80 |
| 6 | AIDS | B20, B21, B22, B23, B24 |
| Reference: Quan H, Sundararajan V, Halfon P, et al. Coding algorithms for defining Comorbidities in ICD-9-CM and ICD-10 administrative data. Med Care. 2005 Nov; 43(11): 1130-9. | | |

**Table S5** Identified pathogens for sepsis cases among COVID-19/seasonal influenza patients and their matched individuals

| **Category of sepsis^*^ or pathogens for bacterial sepsis** | **COVID-19 patients and matched individuals diagnosed with sepsis (N=260)** | | | **Seasonal influenza patients and matched individuals diagnosed with sepsis (N=202)** | | |
| --- | --- | --- | --- | --- | --- | --- |
|  | **COVID-19 patients (N=119)** | **Matched individuals (N=141)** | **P-value**^#^ | **Influenza patients (N=69)** | **Matched individuals (N=133)** | **P-value**^#^ |
| Bacterial sepsis | 76 (63.87%) | 83 (58.87%) | 0.36 | 44 (63.77%) | 80 (60.15%) | 0.62 |
| *Escherichia coli* | 19 (25.00%) | 25 (30.12%) |  | 9 (20.45%) | 29 (36.25%) |  |
| *Other Gram-negative organism(s)* | 8 (10.53%) | 1 (1.20%) |  | 0 (0.00%) | 5 (6.25%) |  |
| *Other staphylococcus* | 5 (6.58%) | 2 (2.41%) |  | 2 (4.55%) | 1 (1.25%) |  |
| *Proteus* | 5 (6.58%) | 1 (1.20%) |  | 2 (4.55%) | 4 (5.00%) |  |
| *Staphylococcus aureus* | 5 (6.58%) | 10 (12.05%) |  | 1 (2.27%) | 8 (10.00%) |  |
| *Pseudomonas* | 4 (5.26%) | 8 (9.64%) |  | 7 (15.91%) | 4 (5.00%) |  |
| *Streptococcus group D* | 4 (5.26%) | 2 (2.41%) |  | 1 (2.27%) | 3 (3.75%) |  |
| *Other streptococcus* | 2 (2.63%) | 4 (4.82%) |  | 2 (4.55%) | 4 (5.00%) |  |
| *Other bacteria(s)* | 24 (31.58%) | 30 (36.14%) |  | 20 (45.45%) | 22 (27.50%) |  |
| Sepsis of unclear pathogen | 42 (35.29%) | 58 (41.13%) |  | 25 (36.23%) | 53 (39.85%) |  |
| Fungal sepsis | 1 (0.84%) | 0 (0.00%) |  | 0 (0.00%) | 0 (0.00%) |  |
| The values were reported as number (%);  ^*^see Additional File 1 Table S3 for the list of ICD-10 codes of each sepsis category; patients assigned with ICD-10 codes of A41.8 or A41.8 only were categorized as unspecified sepsis while those assigned with both A41.8 or A41.9 and other ICD-10 codes were categorized as bacterial sepsis or fungal sepsis (or bacterial and fungal sepsis if any).  ^#^p-values from chi-square test. | | | | | | |
